# A Retrospective Review of Seasonal Patterns of Idiopathic Facial Nerve Paralysis in a Tertiary Care Center

**DOI:** 10.7759/cureus.56075

**Published:** 2024-03-13

**Authors:** Saud M Alfaryan, Fahad Alwadi, Abdullah AlKarni, Abdulaziz K Alaraifi, Khaled S Almolhim, Fahad Alobaid

**Affiliations:** 1 College of Medicine, King Saud Bin Abdulaziz University for Health Sciences, Riyadh, SAU; 2 Otolaryngology - Head and Neck Surgery, King Abdulaziz Medical City Riyadh, Riyadh, SAU; 3 Otolaryngology - Head and Neck Surgery, The Face Institute, Saskatoon, CAN

**Keywords:** bell’s palsy, cold weather, seasonal pattern, facial palsy, facial nerve paralysis

## Abstract

Background

The objective of this retrospective study was to investigate the seasonal patterns of idiopathic facial nerve paralysis, specifically Bell’s palsy, in Riyadh, Saudi Arabia. The study aimed to determine if there is a correlation between cold weather and the incidence of Bell’s palsy, as well as to examine the relationship between age, gender, comorbidities, and the development of the disease.

Methodology

Data were collected from King Abdulaziz Medical City in Riyadh, Saudi Arabia, between 2016 and 2021. Electronic medical records of adult patients diagnosed with idiopathic facial paralysis were reviewed. Patients with facial paralysis caused by known illnesses were excluded. Demographic information, clinical characteristics, and the course of the disease were analyzed using SPSS version 25 (IBM Corp., Armonk, NY, USA).

Results

The study included 136 Bell’s palsy patients, with a mean age of 39.9 years. Males represented 58.1% (79) of the sample, and the right side of the face was more commonly affected in 71 (52.2%) patients. The majority of patients had House-Brackmann grade III (51, 37.5%). The monthly distribution showed a higher number of Bell’s palsy cases during the winter months, particularly December, October, and November, but the seasonal distribution did not yield a statistically significant difference in incidence.

Conclusions

While this study observed a higher incidence of Bell’s palsy during the winter months, it did not establish a statistically significant correlation between cold temperatures and the onset of Bell’s palsy in Riyadh, Saudi Arabia. Furthermore, the study found that Bell’s palsy predominantly affects middle-aged males, and comorbidities did not appear to be significant risk factors for the development of the disease. This research lays the groundwork for future investigations into the relationship between weather and the pathogenesis of Bell’s palsy in the region.

## Introduction

Bell’s palsy is a well-known medical disorder that impairs the facial nerve resulting in a unilateral and asymmetrical facial deformity [1]. Bell’s palsy can be partial weakness or complete paralysis of facial muscles that can last for a few days or, in other cases, remain permanent [2]. Although the pathogenesis of Bell’s palsy is uncertain, the primary risk factors have been identified as cold weather, ischemic, inflammatory, or viral reactivation process (herpes simplex virus 1) [3,4]. Idiopathic facial nerve palsy is the most common form of paralysis throughout the world [5]. It is estimated that in every 100,000 people, between 11 and 40 people suffer from Bell’s palsy [6-8]. Furthermore, it can reach up to 240 people per 100,000, depending on the genetics of certain communities and the climate in which they live [6].

Few researchers in Saudi Arabia have examined the prevalence, etiologies, and incidence of Bell’s palsy in relation to meteorological conditions. From October 2016 to May 2017, a cross-sectional study was conducted in Arar, Saudi Arabia, with 156 patients addressing the prevalent causes of Bell’s palsy [9]. Another prospective research investigated the incidence and relevance of Bell’s palsy in the southwest area of Saudi Arabia, with a total of 321 patients. This research, however, was published in 1997 [10]. Many recent studies in several countries have looked at the association between Bell’s palsy and meteorological parameters such as temperature, wind, and atmospheric pressure. Despite this, there have been no studies about the seasonal pattern of idiopathic facial nerve paralysis in the Middle East, particularly in Saudi Arabia.

Globally, multiple studies have been published to investigate the link between cold weather and Idiopathic Bell’s palsy. One retrospective study conducted in Korea aimed to show the onset of Bell’s palsy in relation to meteorological factors. The study included 385 patients from data obtained from 2007 to 2011 and concluded that high-velocity wind increases the incidence of the disease [11]. In addition, a five-year study from Greece in 2001 examined weather types and meteorological factors in relation to the onset of Bell’s palsy among 171 patients. It was established that there was no correlation between the disease and cold weather [12]. This study aims to investigate the seasonal pattern of idiopathic facial nerve paralysis in a tertiary care center. This study will be able to help future research shed light on the onset of Bell’s palsy to meteorological factors.

## Materials and methods

Study design and subjects

This retrospective study was conducted in King Abdulaziz Medical City, Riyadh, Saudi Arabia. Data were collected from 2016 to 2021. The electronic charts of the patients were reviewed. All adult patients aged 18 years and above who were diagnosed with lower motor neuron facial paralysis were reviewed. Patients who were diagnosed with idiopathic facial paralysis throughout the year were included in the study and those diagnosed with facial paralysis secondary to a known illness were excluded.

Data collection

Patients’ information such as age, sex, and medical comorbidities was obtained from the hospital’s electronic data and entered into an Excel sheet as a data collection form.

Statistical analysis

The data were entered in Microsoft Excel and analyzed using SPSS version 25 (IBM Corp., Armonk, NY, USA). Categorical data were summarized and reported as proportions, while continuous variables were summarized and reported as means and standard deviations (SDs). The differences between the study groups regarding the clinical manifestations and the course of the disease were compared using the chi-square test. A p-value <0.05 was considered statistically significant.

## Results

The study included 136 patients with Bell’s palsy. The mean age of our patients was 39.9 (±17.2) years, and 79 (58.1%) patients in our sample were males. The right side of the face was more commonly involved than the left side in 71 (52.2%) and 65 (47.8%) patients, respectively. House-Brackmann grade III was the most reported grade in 51 (37.5%) patients, followed by grades IV, II, and V. Table 1 demonstrates patients’ demographic and clinical characteristics.

**Table 1:** Demographic and clinical characteristics of all study participants.

Variables	Statistics (n = 136)
Age (mean ± SD)	39.9 (±17.2)
Gender, N (%)	
Male	79 (58.1%)
Female	57 (41.9%)
Comorbidities, N (%)	
Yes	58 (42.6%)
No	78 (57.4%)
Facial side, N (%)	
Right	71 (52.2%)
Left	65 (47.8%)
Grade of paralysis, N (%)	
2	23 (16.9%)
3	51 (37.5%)
4	47 (34.6%)
5	15 (11.0%)

Table 2 illustrates the monthly distribution of all cases with Bell’s palsy. The most common months with documented cases were December (15.4%), October (13.2%), and November (10.3%). On the other hand, February and June were the months with fewer documented cases at 5.1% in both months.

**Table 2:** Monthly distribution of all cases with Bell’s palsy.

Month	Statistics (n = 136)
January, N (%)	9 (6.6%)
February, N (%)	7 (5.1%)
March, N (%)	12 (8.8%)
April, N (%)	8 (5.9%)
May, N (%)	9 (6.6%)
June, N (%)	7 (5.1%)
July, N (%)	11 (8.1%)
August, N (%)	9 (6.6%)
September, N (%)	11 (8.1%)
October, N (%)	18 (13.2%)
November, N (%)	14 (10.3%)
December, N (%)	21 (15.4%)

**Figure 1:**
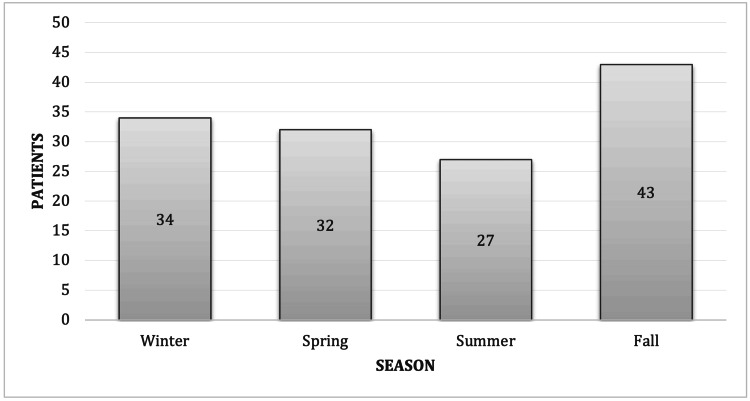
Seasonal distribution of all cases with Bell’s palsy (p = 0.268).

Figure 1 illustrates the seasonal distribution of all cases with Bell’s palsy. Fall was the most common season with documented cases (31.6%), while summer had the least reported cases (19.9%). However, there was no significant difference in the incidence of Bell’s palsy in the four seasons (p = 0.268).

## Discussion

The goal of this retrospective research is to identify whether seasonal variations and cold temperatures can contribute to idiopathic facial nerve paralysis and highlight the relationship between age, sex, or comorbidities and the pathogenesis of the disease.

Our study demonstrated a high number of Bell’s palsy cases during winter compared to summer, which showed a decrease in the number of Bell’s palsy cases. However, the result of the study is not statistically significant. The shift in temperature between seasons, especially around August to October, has been shown to contribute to an increase in Bell’s palsy cases in the hospital, which may indicate that not only cold temperatures can cause Bell’s palsy but also the rapid change in weather may potentially trigger the disease. According to a recent article published in 2022, exposure to cold weather was found to be a significant risk factor for Bell’s palsy in the Qurrayat region of Saudi Arabia [13]. Located in the northern region of Saudi Arabia, Qurrayat shares a similar climate with Riyadh, but temperatures drop below 0°C during winter. Our results support previous evidence linking cold weather to the incidence of Bell’s palsy. However, females were predominantly affected by Bell’s palsy in the Qurruyat region, contrary to our findings. From this study, medically free middle-aged males were more prone to suffer from Bell’s palsy compared to their female counterparts. Moreover, pre-existing conditions ranging from autoimmune, neurological, cardiovascular, or previous injuries did not play a role in idiopathic facial nerve paralysis. A study was performed in Italy to investigate the relationship between hypertension and diabetes with Bell’s palsy [14]. The study involved 381 cases and found no significant difference in the incidence of Bell’s palsy due to hypertension or diabetes, which reinforces our results.

Factors other than meteorological parameters can play a role in the incidence of Bell’s palsy. A nested case-controlled study identified a correlation between air pollution, notably NO_2_ exposure, and the risk of Bell’s palsy, indicating that environmental factors beyond mere climatic conditions may play a pivotal role in the etiology of Bell’s palsy. The study highlighted that exposure to NO_2_ was significantly elevated in Bell’s palsy patients compared to controls during the 60 days preceding the onset of the condition [15]. A study conducted from January 1992 to June 1996 retrospectively reviewed Bell’s palsy cases where the onset date was known. Daily data on temperature and air pollutants (including particle count and levels of SO_2_, CO, O_3_, NO_3_, NO, and CH_4_) were recorded by the Spanish National Service of Meteorology. The study found a correlation between lower temperatures and increased Bell’s palsy incidents, but no link between atmospheric pressure, air pollutants, and the condition was established [16].

Although this is the first study to be conducted in Riyadh, Saudi Arabia, the study has some limitations. Multiple meteorological factors other than weather can contribute to an increased risk of Bell’s palsy and should be taken into consideration. Humidity, wind speed, and atmospheric pressure play a role in the development of Bell’s palsy, as seen in a multicenter cross-over study done in Berlin, Germany. The study findings concluded that acute atmospheric pressure changes in the last 24 hours before Bell’s palsy are linked to a 35% increase in cases [17]. Additionally, only 136 patients were included in the study who fit the inclusion criteria. This limitation suggests the need for multicenter studies to be conducted in the future with a larger sample size.

This retrospective study has several strengths. The utilization of electronic medical records provided comprehensive past medical history, referrals, and follow-ups for patients, thereby ensuring the accuracy of the information gathered. To eliminate bias among data collectors, each researcher checked the data of the 136 patients included in the study. The date of first admission, the department they were admitted in, past medical history, follow-ups, and medications given were documented from electronic medical records. Additionally, the study is the first stepping stone to investigate the pathogenesis of Bell’s palsy regarding weather in Riyadh, Saudi Arabia.

## Conclusions

The study has documented a rise in Bell’s palsy cases during winter, nonetheless, it is not scientifically significant to support the correlation between cold temperatures and the incidence of Bell’s palsy. Additionally, the study found that Bell’s palsy most commonly affects individuals at an average age of 39.9 years, with males being more susceptible to the disease. Lastly, comorbidities were not shown to be a risk factor for the development of Bell’s palsy.
